# Evaluation of the diagnostic performance of colposcopy in the detection of cervical high-grade squamous intraepithelial lesions among women with transformation zone type 3

**DOI:** 10.1186/s12885-024-12156-2

**Published:** 2024-03-26

**Authors:** Xiaoxiao Li, Yunzhi Zhao, Fenfen Xiang, Xinpei Zhang, Zixi Chen, Mengzhe Zhang, Xiangdong Kang, Rong Wu

**Affiliations:** 1 Department of Laboratory Medicine, Putuo Hospital, Shanghai University of Traditional Chinese Medicine, Shanghai, China; 2 Department of Obstetrics and Gynecology, Putuo Hospital, Shanghai University of Traditional Chinese Medicine, Shanghai, China

**Keywords:** Colposcopy, Cervical biopsy, Accuracy, High-grade squamous intraepithelial lesions, Cervical cancer

## Abstract

**Background:**

Inaccurate colposcopy diagnosis may lead to inappropriate management and increase the incidence of cervical cancer. This study aimed to evaluate the diagnostic accuracy of colposcopy in the detection of histologic cervical intraepithelial neoplasia grade 2 or worse (CIN2+) in women with transformation zone type 3 (TZ3).

**Methods:**

Records from 764 patients with TZ3 who underwent colposcopy-directed biopsy and/or endocervical curettage in Putuo Hospital China between February 2020 and March 2023 were retrospectively collected. Colposcopy was carried out based on 2011 International Federation of Cervical Pathology and Colposcopy (IFCPC) and Colposcopy nomenclature. The diagnostic performance of colposcopy for identifying CIN2 + was evaluated compared with biopsies. The Kappa and McNemar tests were used to perform statistical analyses.

**Results:**

Among the study population, 11.0% had pathologic CIN2+. The relative sensitivity, specificity, positive predictive value (PPV), and negative predictive value (NPV) of colposcopy for histologic CIN2 + were 51.2%, 96.5%, 64.2% and 94.1%, respectively. The senior colposcopists (80.6%) had a higher colposcopic accuracy to diagnose histologic CIN2 + than junior colposcopists (68.6%). In subgroup analyses, age group ≥ 60 years (70.3%) showed lowest diagnostic accuracy when compared with age groups of < 45 years (84.4%) and 45–59 years (74.9%).

**Conclusion:**

Our findings suggest an increased risk of diagnostic inaccuracy of colposcopy in identifying CIN2 + in those ≥ 60 years of age with TZ3, and the accuracy of colposcopy is required to be further improved.

## Introduction

Cervical cancer is the fourth most common cancer in women worldwide, and eighth most common cause of tumor-related mortality in Chinese women, with 119,300 new cases and 59,060 deaths in 2020 [1]. Screening with HPV testing and/or cytology, offers an opportunity to identify women who are at a higher risk of precancerous conditions [2, 3], whereas colposcopy and biopsies are important parts of the diagnostic workup [4]. It has been reported that older women have higher cervical cancer incidence and mortality rates than younger women [5, 6]. Moreover, older women are more commonly diagnosed with advanced-stage disease squamous cell carcinoma and adenocarcinoma) [7] and have a poorer prognosis [8]. This may be due to insufficient screening, screening failure, diagnostic difficulties and insufficient follow-up.

Colposcopy is an indispensable tool for early detection, and accurate use can benefit women by reducing the frequency of unnecessary biopsies, conization surgeries, as well as the frequency of cauterization treatments for cervical erosion [9]. This means, there is a great amount of avoidable stress caused by diagnostic inaccuracies and discrepancies between colposcopic and pathological diagnosis [10]. Many factors can affect colposcopic accuracy, such as colposcopists^,^ skills, screening results, transformation zone (TZ) type and number of biopsies [11, 12]. Management difficulties arise when the TZ is entirely within the endocervical canal, namely transformation zone type 3 (TZ3). Nearly 20% of colposcopic assessments are inadequate due to a TZ3 [13]. Endocervical canal curettage (ECC) which provides fragments of squamous epithelium from inside the cervical canal is generally used in clinical practice for adjunctive biopsy of women with TZ3 at colposcopy [14], and it has been considered should be performed for patients with ASC-H/HSIL cytology, 16/18 HPV infection and those with high-grade colposcopic impressions [15]. Some investigators have reported that adding referral screening results to colposcopic examinations can improve CIN2 + detection, especially for women with TZ3 lesions [16, 17]. Therefore, it remains necessary to identify and assess potential causes of colposcopic inaccuracies and understand diversity and variance in order to reduce unnecessary stress caused and improve outcomes.

The primary aim of this study was to investigate discrepancies between colposcopic and cervical biopsy in women with a TZ3, and the performance of colposcopy for identifying CIN2 + were compared with cervical biopsies.

## Materials and methods

### Study population

This is a retrospective cohort of women who underwent colposcopic examination as a result of positive screening tests and/or abnormal clinical symptoms between February 2020 and March 2023 at Putuo Hospital, a large tertiary center in Shanghai, China. All women were managed by a select-and-treat approach. If an abnormal TZ was identified, a colposcopy-directed biopsy was performed on the abnormal area. When the TZ was not completely visible or no colposcopic abnormalities were identified, an ECC with a Novak curette was performed, if necessary. Of note, the collection of ECC is not routinely recommended in the guidelines but may be performed depending on the colposcopist^,^s performance.

The TZ is partially or fully located in the endocervix with no visible squamocolumnar junction (SCJ) corresponding to TZ3. Eligible for inclusion in this study were women who had TZ type 3 according to 2011 International Federation of Cervical Pathology and Colposcopy (IFCPC) and Colposcopy nomenclature [18]. The colposcopists^,^ clinical findings during colposcopy were compared with the final histological results from the punch biopsies and/or endocervical curettage. Women were excluded if they had a hysterectomy or previous excisional treatment for CIN (including cold knife conization, loop electrosurgical excision procedure and cervical laser conization) or received estrogen medical treatment, intended to get pregnant, or underwent follow-up for a previously diagnosed CIN. Moreover, women who underwent colposcopy but had no histologic diagnosis were also excluded. All data, including age, HPV screening result, cytology, indication for colposcopy, TZ types, colposcopic impressions, colposcopist’s level and histological results were recorded prospectively for further research. This study was conducted in accordance with the Declaration of Helsinki and was approved by the Institution Review Board of Putuo Hospital, Shanghai University of Traditional Chinese Medicine. As the retrospective analysis was based on anonymized data, the need for individual informed consent was waived.

### HPV and liquid-based cytology testing

HPV DNA testing was performed using the clinically validated cobas^@^4800 platform (Roche Diagnostic, USA) [19] which detects HPV16, HPV18 and a pool of 12 other high-risk HPV genotypes (HPV31/33/35/39/45/51/52/56/58/59/66/68). Liquid-based cytology was performed by introducing a cervical plastic brush into the external cavity and scraping cells from the exocervix and endocervix, and then placed on a smear slide and fixed. Cytology slide results were classified according to the Bethesda grading system (2014) [20], including no intraepithelial lesions or malignancy (NILM), atypical squamous cells of undetermined significance (ASC-US), low-grade squamous intraepithelial lesion (LSIL), atypical glandular cells of undetermined significance (AGUS), atypical squamous cells cannot exclude high-grade squamous intraepithelial lesion (ASC-H), high-grade squamous intraepithelial lesion (HSIL) or carcinoma.

### Colposcopy and histology diagnosis

All colposcopies were performed by gynecologists using an electrionic colposcope (EDAN C6 HD) after preparing the cervix with 5% acetic acid and Lugol’s iodine solution. Colposcopic features assessed in the study included the presence of acetowhite epithelium and its characteristics. The colposcopic diagnostic results according to the 2011 colposcopic terminology of IFCPC included: normal, low-grade lesions, high-grade lesions and suspicious for invasion colposcopic findings [21]. During the period of this retrospective analysis, the team of cervix consisted of 10 colposcopists with various degrees of clinical experience and training. Briefly, colposcopists with more than 10 years of working experience were defined as senior colposcopists, and others were categorized as junior colposcopists. Histopathological outcomes were graded according to World Health Organization (WHO) terminology: normal, cervical intraepithelial neoplasia grade 1 (CIN1), cervical intraepithelial neoplasia grade 2 (CIN2), cervical intraepithelial neoplasia grade 3 (CIN3) and invasive carcinoma [22]. The histopathologic results were taken as the gold standard in the study. When analyzing biopsies and/or endocervical curettage together, the worst grade of lesion was considered as the final histological diagnosis. The accordance was the percentage of women diagnosed by colposcopy and histopathological findings. Over-diagnosis was considered to be present when the histopathological findings are less severe than those obtained during colposcopy. Under-diagnosis was considered to have occurred when histopathological findings highlighted more advanced lesions than colposcopic findings.

### Statistical analysis

Descriptive statistics were used to describe clinical characteristics of the study population. The diagnostic performance of colposcopy for detecting CIN2 + was presented in the form of a 2 × 2 table. The agreement between colposcopic findings and histopathological diagnosis was evaluated by Cohen’s kappa (κ) coefficient and Chi-square test at a significance level of 5%. The strength of agreement was judged by the criteria as follows: 0-0.2 as slight, 0.21–0.40 as fair, 041 − 0.60 as moderate, 0.61–0.80 as substantial, and 0.81-1.00 as almost perfect agreement. The exact McNemar’s test was performed to evaluate the diagnostic difference of colposcopy and biopsy to differentiate CIN2+. Relative sensitivity, relative specificity, balanced accuracy, false positive rate (FPR), false negative rate (FNR), positive predictive value (PPV), and negative predictive value (NPV) were used to assess the diagnostic performance of colposcopy for CIN2+. The 95% confidence interval (CI) was estimated. Data analysis was performed using Excel (version 2010) and SPSS software (version 22.0). A p-value < 0.05 was considered statistically significant.

## Results

### Clinical characteristics of study population

**Fig. 1:**
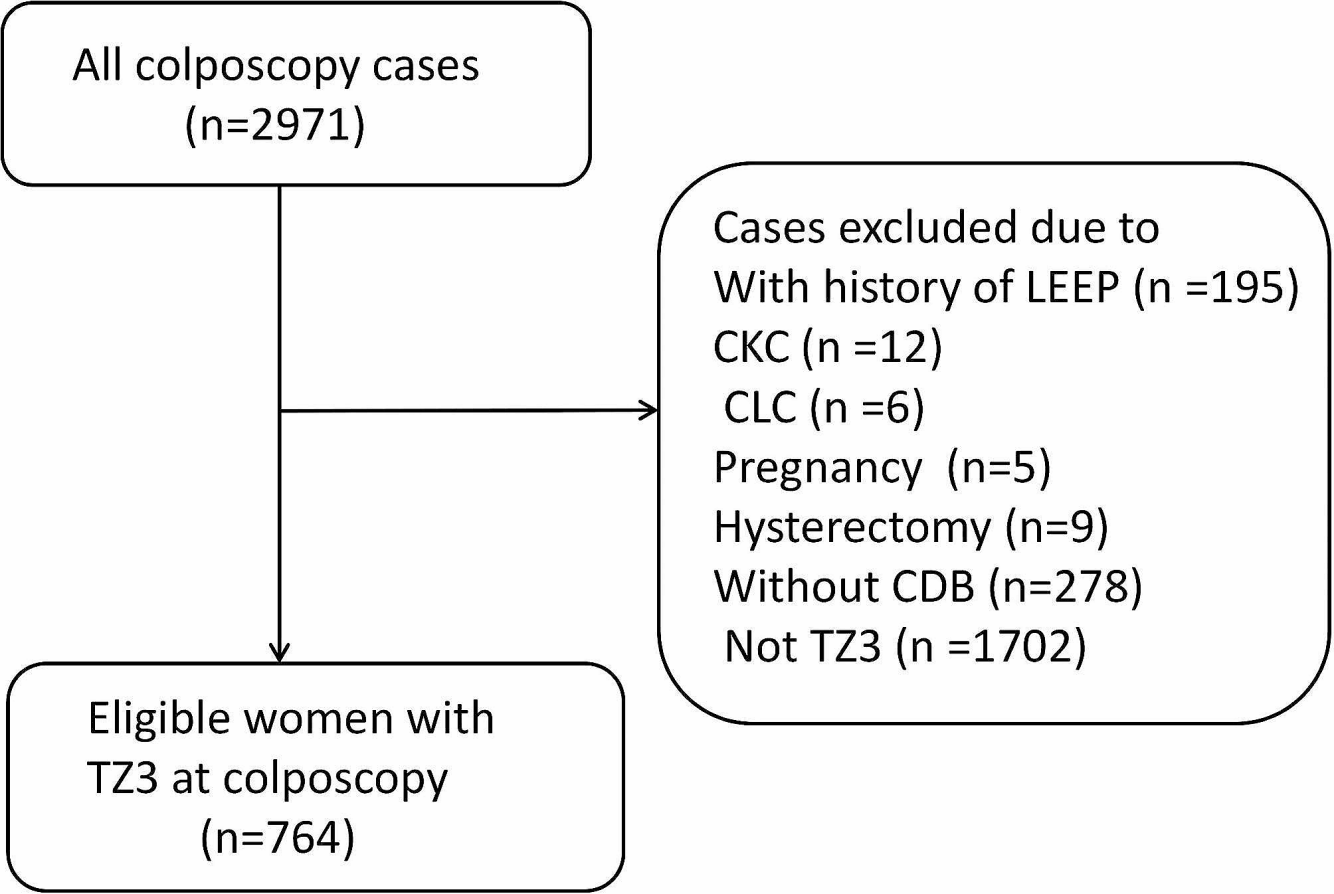
Flowchart illustrating the selection of study population. TZ, transformation zone; LEEP, loop electrosurgical excision procedure; CKC, cold-knife conization; CLC, cervical laser conization; CDB, colposcopy-directed biopsy

The flowchart selection of study population is depicted in Fig. 1. In total, 764 women with TZ3 who underwent colposcopy-directed biopsy were included in this analysis. Detailed patient characteristics and relevant clinical findings are provided in Table 1. The mean age of the study women was 52.9 ± 11.8 years (range, 20–84 years). Nearly, 77.0% of the patients were 45 years or more of age. Among them, 91 patients (11.9%) had undergone colposcopy due to abnormal clinical signs. Whereas abnormal screening test results (88.1%) remained the most common reason for colposcopy. 724 women (94.8%) had undergone primary HPV screening and 615 (80.5%) women had high-risk genotypes. The most common cytology results were NILM (70.0%), followed by LSIL/ASCUS/AGUS (23.0%) and HSIL+/ASC-H (3.6%). For colposcopy diagnosis, the proportions of low-grade, high-grade and suspicious cancer were 18.2%, 5.6% and 3.2%, respectively. The overall incidence of pathologic CIN2 + in women with TZ3 was 11.0%.

**Table 1:** Description of the study population

Characteristics	All (*n* = 764)
**Age (years)**	
Mean ± SD	52.9 ± 11.8
< 45	176 (23.0%)
45–59	321 (42.0%)
≥ 60	267 (35.0%)
**HPV status**	
Unknown or not performed	40 (5.2%)
Negative	109 (14.3%)
HPV16/18	162 (21.2%)
Non-16/18 HR-HPV	453 (59.3%)
**Cytology results**	
Unknown or not performed	26 (3.4%)
NILM	535 (70.0%)
LSIL/ASCUS/AGUS	176 (23.0%)
HSIL+/ASC-H	27 (3.6%)
**Colposcopic findings**	
Normal	558 (73.0%)
Low-grade lesion	139 (18.2%)
High-grade lesion	43 (5.6%)
Suspicious of cancer	24 (3.2%)
**Histology results**	
<CIN2	680 (89.0%)
Normal/ cervicitis	527 (69.0%)
CIN1	153 (20.0%)
CIN2+	84 (11.0%)
CIN2/CIN3	62 (8.1%)
SqCC	18 (2.4%)
AC	4 (0.5%)
**Colposcopist**	
Junior Senior **Indication for colposcopy**	525 (68.7%) 239 (31.3%)
Abnormal clinical symptoms Abnormal vaginal bleeding Abnormal vaginal discharge Post-coital bleeding Unusual appearance of the cervix Abnormal screening results	91 (11.9%) 31 (4.1%) 19 (2.5%) 25 (3.3%) 16 (2.1%) 673 (88.1%)

NILM, negative for intraepithelial lesion or malignancy; LSIL, low-grade squamous intraepithelial lesion; ASCUS, atypical squamous cells of undetermined significance; AGUS, atypical glandular cells of undetermined significance; ASC-H, atypical squamous cells-cannot exclude HSIL; HSIL, high-grade squamous intraepithelial lesion; CIN, cervical intraepithelial neoplasia; CIN2+, cervical intraepithelial neoplasia grade 2 or worse; CIN2, cervical intraepithelial neoplasia grade 2; CIN3, cervical intraepithelial neoplasia grade 3; SD, standard deviation; SqCC, squamous cell carcinoma; AC, adenocarcinoma

### Consistency between colposcopic diagnosis and histopathology

**Fig. 2:**
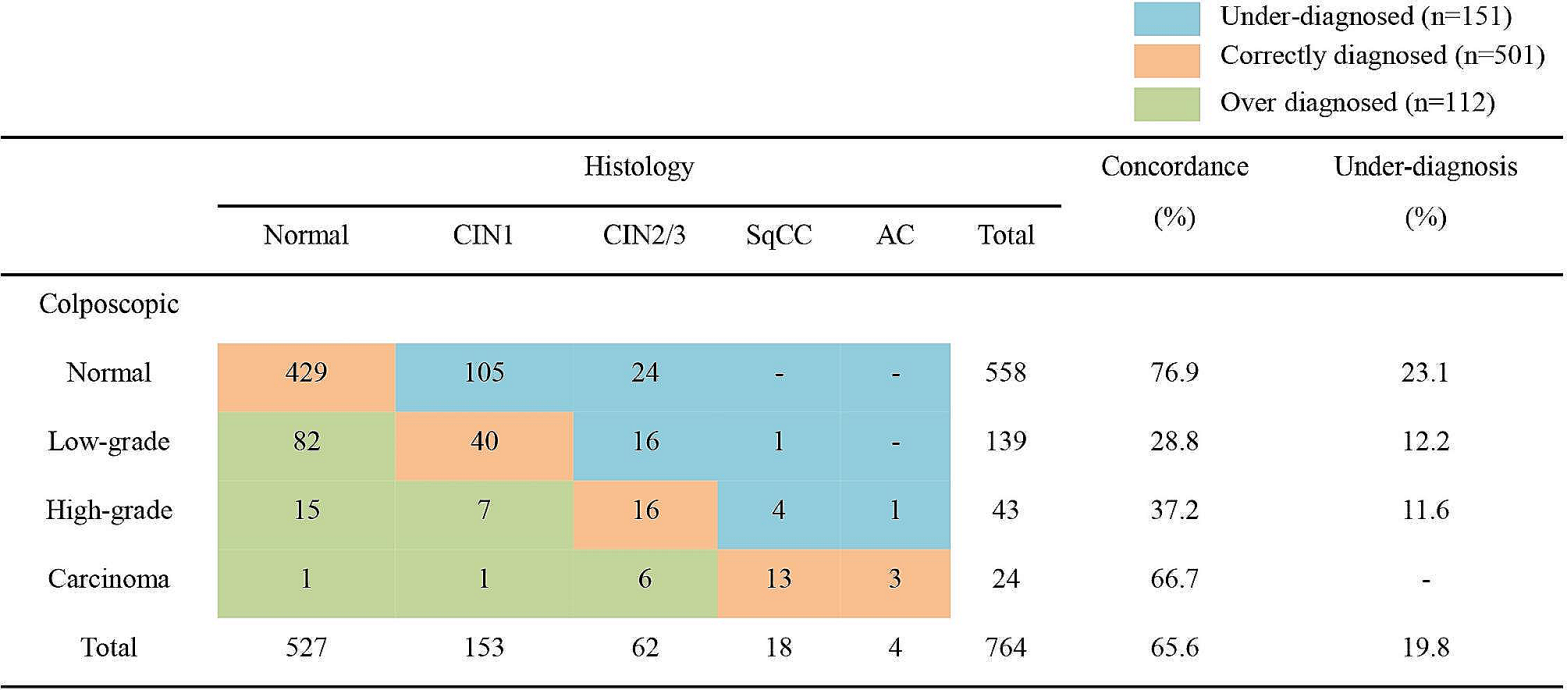
Comparison of results between colposcopic findings and histology. CIN1, cervical intraepithelial neoplasia grade 1; CIN2, cervical intraepithelial neoplasia grade 2; CIN3, cervical intraepithelial neoplasia grade 3; SqCC, squamous cell carcinoma; AC, adenocarcinoma

We compared the detailed consistency between the colposcopic assessment and the histopathologic results (Fig. 2). The overall concordance rate was 65.6% (501/764). Under-diagnosed cases were observed in 19.8% (151/764). Among 151 under-diagnosed cases, 105 (69.5%), 40 (26.5%), 5 (3.3%), and 1 (0.7%) were finally diagnosed with CIN1, CIN2/3, SqCC and AC, respectively. Specifically, among 139 cases that had been diagnosed with low-grade impressions by colposcopy, 16 cases (11.5%) were correctly diagnosed with CIN2/3 and 1 case (0.7%) with SqCC.

### Diagnostic performance of colposcopy in the detection of CIN2+

**Fig. 3:**
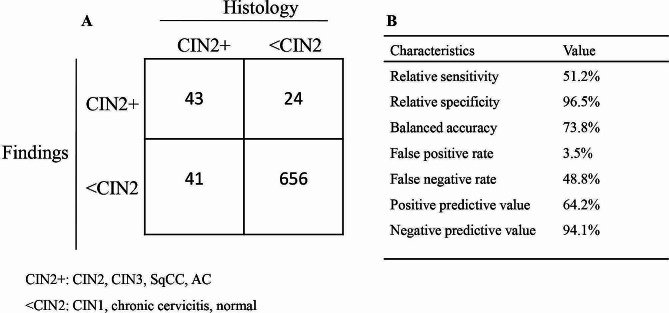
Diagnostic performance of colposcopy in patients with TZ3.(**A**) 2 × 2 contingency table, (**B**) value for each parameter.TZ, transformation zone; CIN, cervical intraepithelial neoplasia; SqCC, squamous cell carcinoma; AC, adenocarcinoma

Considering the histologic results as the gold standard, we evaluated the diagnostic performance of colposcopy for identifying CIN2 + with the following results: relative sensitivity, 51.2%; relative specificity, 96.5%; balanced accuracy, 73.8%; PPV, 64.2% and NPV, 94.1% (Fig. 3). Cohen’s κ coefficient for colposcopy and biopsy was 0.523, indicating moderate agreement. However, the exact McNemar’s test revealed that colposcopy was significantly different in the diagnosis of CIN2 + when compared to biopsy in all women with TZ3 (*p* = 0.047). We also performed subgroup analysis according to colposcopists^,^ experience. Cohen’s κ coefficients for colposcopy and biopsy in subgroup of junior and senior colposcopists were 0.399 (fair agreement) and 0.685 (substantial agreement), respectively. The specificity between senior and junior colposcopists was comparable. Whereas, the relative sensitivity of senior (63.2%) was higher than junior (41.3%) colposcopists (Table 2).

**Table 2:** Diagnostic performance of colposcopy in detecting CIN2 + among patients with transformation zone type 3

Group	Sensitivity (95%CI)	Specificity (95%CI)	Accuracy (95%CI)	FPR (95%CI)	FNR (95%CI)	PPV (95%CI)	NPV (95%CI)
Junior	41.3% (27.1–55.5%)	95.8% (94.0-97.6%)	68.6% (64.6–72.5%)	4.2% (2.4–5.9%)	58.7% (44.5–72.9%)	48.7% (33.0-64.4%)	94.4% (92.4–96.5%)
Senior	63.2% (47.8–78.5%)	98.0% (96.1–99.9%)	80.6% (75.6–85.6%)	2.0% (0.1–3.9%)	36.8% (21.5–52.2%)	85.7% (72.8–98.7%)	93.4% (90.0-96.7%)

CIN2+, cervical intraepithelial neoplasia grade 2 or worse; FPR, false positive rate; FNR, false negative rate; PPV, positive predictive value; NPV, negative predictive value

### Age-specific subgroup analysis

**Fig. 4:**
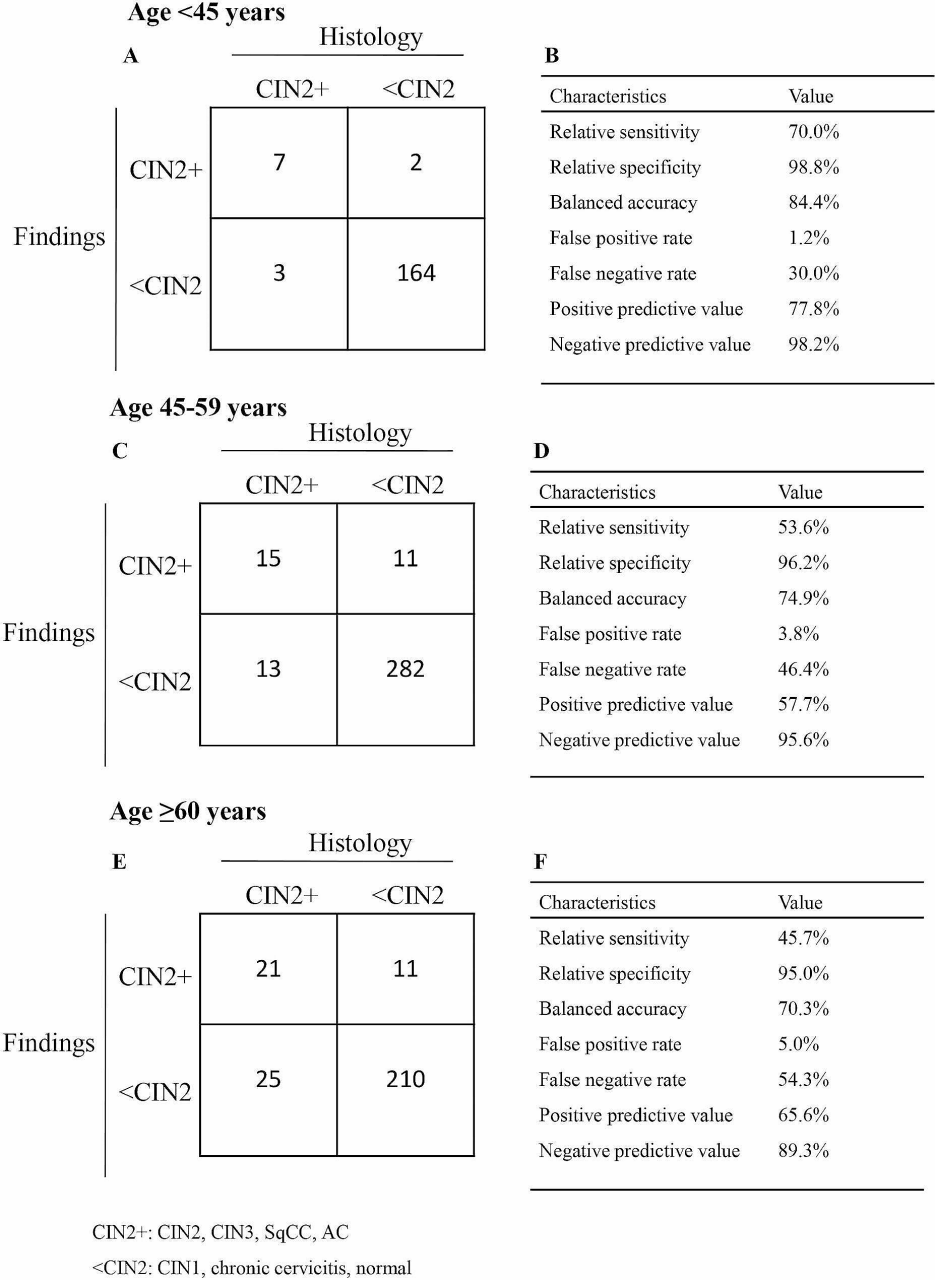
Diagnostic performance of colposcopy in age-specific subgroups including patients aged < 45 years, 45–59 years, and ≥ 60 years. (**A, C, E**) 2 × 2 contingency table, (**B, D, F**) value for each parameter. TZ, transformation zone; CIN, cervical intraepithelial neoplasia; SqCC, squamous cell carcinoma; AC, adenocarcinoma

The performance of colposcopy in the detection of histologic CIN2 + in different age groups is shown in Fig. 4. In the younger group < 45 years, the diagnostic performance of colposcopy for identifying CIN2 + was as follows: relative sensitivity, 70.0%; relative specificity, 98.8%; balanced accuracy, 84.4%; PPV, 77.8% and NPV, 98.2% (Fig. 4AB). Cohen’s κ coefficient for colposcopy and biopsy was 0.721, indicating substantial agreement. The overall rates for under-, correctly- and over-diagnosed cases were 16.5%, 72.2% and 11.3%, respectively (Table 3).

**Table 3:** Diagnostic accuracy of colposcopy according to the patients’ age group

	Overall	< 45 years	45–59 years	≥ 60 years
Under-diagnosis	19.8% (151/764)	16.5% (29/176)	21.2% (68/321)	20.3% (54/267)
Accordance	65.6% (501/764)	72.2% (127/176)	63.2% (203/321)	64.0% (171/267)
Over-diagnosis	14.7% (112/764)	11.3% (20/176)	15.6% (50/321)	15.7% (42/267)

In the middle subgroup of patients aged 45–59 years, the diagnostic performance of colposcopy for identifying CIN2 + was as follows: relative sensitivity, 53.6%; relative specificity, 96.2%; balanced accuracy, 74.9%; PPV, 57.7% and NPV, 95.6% (Fig. 4CD). Cohen’s κ coefficient for colposcopy and biopsy was 0.513, indicating moderate agreement. The overall rates for under-, correctly- and over-diagnosed cases were 21.2%, 63.2% and 15.6%, respectively (Table 3).

In the older group ≥ 60 years, the diagnostic performance of colposcopy for identifying CIN2 + was as follows: relative sensitivity, 45.7%; relative specificity, 95.0%; balanced accuracy, 70.3%; PPV, 65.6% and NPV, 89.3% (Fig. 4EF). Cohen’s κ coefficients for colposcopy and biopsy was 0.462, indicating moderate agreement. The overall rates for under-, correctly- and over-diagnosed cases were 20.3%, 64.0% and 15.7%, respectively (Table 3).

## Discussion

This cross-sectional study included 764 colposcopies with histology. The risk of CIN2 + among these patients with TZ3 was 11.0%. The diagnostic accuracy in the detection of histologic CIN2 + in women with TZ3 is quite challenging, particularly in the elderly. Women with TZ3 were the most commonly encountered among women older than 50 years (70%) in a prospective multicenter study by Luyten et al. [23]. squamocolumnar junction may be invisible in perimenopausal women, and cervical lesions may extend into the endocervical canal, thus rendering colposcopic examination unsatisfactory. A recent observational study conducted in Denmark also revealed that most women (74.9%) have TZ3 at colposcopy, and 20.0% of women had CIN2 + diagnosed among women aged ≥ 69 referred to colposcopy due to an HPV-positive screening test. The findings also suggested a potential risk of underdiagnosis of CIN2 + in older postmenopausal women referred to colposcopy [24]. Additionally, Gustafson et al. [11] found that CIN2 + detection is underestimated when comparing the proportion of CIN2 + in cervical biopsies with that in large loop excision of the transformation zone (LLETZ) specimens in women aged ≥ 45 years with TZ3.

In this study, Our results revealed a relatively lower balanced accuracy (73.8%), relative sensitivity (51.2%), and raise specificity (96.5%) when CIN2 + as the threshold, which was comparable to studies reported in Bangladesh by Ara et al. (sensitivity, 50%; specificity, 94.0%) [25] and in China by Ruan et al. (sensitivity, 56.29%; specificity, 93.82%) [26]. A similar study conducted in Bhutan found the sensitivity of colposcopy to detect CIN2 + was 66.67%, and accuracy was 73.73% [27]. In a study also done in China, the sensitivity, specificity, and accuracy of colposcopy to diagnose histologic CIN2 + were 70.2%, 75.1% and 72.9%, respectively [16]. A recent meta-analysis showed that colposcopic accuracy for detecting CIN2 + was 89%, with combined sensitivity and specificity were 68% and 93%, respectively [28]. The sensitivity of colposcopic impressions ranged from 29 to 100% and the specificity from 12 to 88% based on 11 studies [29].

Colposcopy is a highly subjective examination method, the performance of colposcopy in cervical pathological diagnosis varies greatly among observers, which depends on the duration of the experience of colposcopists [27]. In our previous study, the diagnostic performance of colposcopy in the diagnosis of CIN2 + by senior and junior colposcopists was comparable in women with oncogenic HPV regardless of TZ type [30]. However, herein subgroup according to colposcopists^,^ experience, we found the relative sensitivity (63.2% versus 41.3%) and accuracy (80.6% versus 68.6%) of senior colposcopists to identify CIN2 + were significantly higher than those of junior colposcopists. The specificity (98.0% versus 95.8%) of seniors was slightly higher than juniors. Dorji et al. [27] also found the sensitivity (80.00% versus 59.46%) of senior colposcopists was higher than junior colposcopists. However, senior colposcopists had lower specificity (71.07% versus 76.72%) and almost comparable accuracy (72.60% versus 72.55%). In a similar study done in Germany, the colposcopic sensitivity (86.0% versus 70.2%) of senior colposcopists to diagnose CIN2 + lesions was higher than junior colposcopists, with lower specificity (65.1% versus 68.6%) [31]. One possible explanation might be the fact that young colposcopists lack of course and some amount of knowledge and skills acquired over the years. Our data underline the need for qualified staff including standardized colposcopy steps, regular updated courses, regular supervision and quality assurance measures, especially for junior colposcopists.

In subgroup analysis according to patients^,^ age, we found the diagnostic accuracy of colposcopy for identifying CIN2 + decreased as age increased with the balanced accuracy of colposcopy in age group < 45 years, 45–59 years and ≥ 60 years being 84.4%, 74.9% and 70.3%, respectively. A recent retrospective study reported the accuracy rates for detecting HSIL which were 65.67% (age ≤ 30 years ), 71.12% (31–45 years) and 60.43% (> 45 years) [32]. Some studies have also observed that the diagnostic accuracy of colposcopy-guided biopsy for identifying HSIL + decreased with increasing age. For example, Kim et al. [33] found that the diagnostic accuracy of colposcopy-guided biopsy in age groups < 35 years was 81.0%, 74.4% for 35–50 years and 68.8% for those patients aged ≥ 50 years. Stuebs et al. [17] also reported similar trends in the accuracy rates for detecting HSILs. The authors themselves postulated that relatively poor diagnostic performance for identifying HSIL + in women ≥ 50 years might be related to postmenopause, unidentifiable SCJ or cervical lesions that are not well visualized with colposcopy. CIN2 + cases were more likely to be missed among older women and therefore should be considered more carefully during clinical consultations.

A major strength of this study was the use of real-world data from a number of women with TZ3 at colposcopy. Our study contributed results from a highly unique group of women attending colposcopy which provided baseline data, and way forward for improvement. However, there are several limitations that should be considered. First, although we selected consecutive patients with clearly defined eligibility criteria, inevitable issues might arise in retrospective studies such as selection bias. For example, women with TZ3 but without histology diagnosis were excluded, which will yield inherent bias. Second, as a single-institution study, the sample size might be insufficient, and larger numbers are needed to make the results more robust. Third, the biopsy specimens were taken only from suspicious lesions without comparable control specimens. Finally, we have only studied colposcopic accuracy for detecting CIN2+, the data required to discern differences between CIN2+, CIN3 + and cervical cancer are also meaningful.

## Conclusions

In conclusion, the overall diagnostic accuracy of colposcopy and the consistency between colposcopy and histology in our study were comparable to previous studies, but further improvement was required. The relative sensitivity and balanced accuracy among junior colposcopists were lower than senior colposcopists. Moreover, diagnostic inaccuracies of colposcopy were magnified in those ≥ 60 years old. Future measures towards improving the performance of colposcopy such as using a reasonable scoring system and standard diagnostic criteria are still warranted.

## Data Availability

The datasets used and/or analysed during the current study are available from the corresponding author on reasonable request.
